# Osseointegrated prosthesis for patients with an amputation: Multidisciplinary team approach in the Netherlands

**DOI:** 10.1007/s00113-016-0302-1

**Published:** 2017-01-17

**Authors:** J. P. M. Frölke, R. A. Leijendekkers, H. van de Meent

**Affiliations:** 1 Dept. of Surgery, Radboud university medical center, Nijmegen, The Netherlands; 2 Dept. of Orthopedics, Radboud university medical center, Nijmegen, The Netherlands; 3 Dept. of Rehab. Medicine, Radboud university medical center, Nijmegen, The Netherlands; 4 (Orthopaedic) Trauma surgeon, Radboud university medical center, Geert Grooteplein Zuid 10 (route 618), 6525 GA Nijmegen, The Netherlands

**Keywords:** Amputation, Osseointegration, Bone-anchored prosthesis, Rehabilitation, Multidisciplinary approach, Amputation, Osseointegration, Knochenverankerte Prothese, Rehabilitation, Multidisziplinärer Ansatz

## Abstract

This article reviews the development of multidisciplinary osseointegration treatment in the Netherlands since its start in 2009. People experiencing limitations due to their socket prosthesis after a leg amputation present to the Radboud University Nijmegen Medical Centre for an osseointegration implant or “bone-anchored” prosthesis. In this article we share our experience with the first 100 patients regarding referral pattern, selection criteria, available osseointegration systems, preoperative planning, surgical treatment, the rehabilitation protocol, outcome measurement, revision surgery, and future developments.

## Zusammenfassung

Der Beitrag fasst die Entwicklung der multidisziplinären Osseointegrationsbehandlung in den Niederlanden seit ihren Anfängen im Jahr 2009 zusammen. Patienten nach Beinamputation, die durch ihre stumpfumfassende Prothese eingeschränkt sind, stellen sich im Radboud University Nijmegen Medical Centre vor, um ein osseointegrierendes Implantat oder eine „knochenverankerte“ Prothese zu erhalten. Wir berichten über unsere Erfahrungen mit den ersten 100 Patienten. Thematisiert werden Einweisungsmuster, Auswahlkriterien, verfügbare Osseointegrationssysteme, die präoperative Planung, die operative Behandlung, das Rehabilitationsprotokoll, Ergebnissmessung, Revisionsoperationen und zukünftige Entwicklungen.

## Referral and assessment

People with a lower limb amputation who encounter problems with their socket prosthesis present to our clinic via different routes. When the orthopedic technicians are unable to make a suitable socket, they usually refer the person to the treating rehabilitation consultant. All rehabilitation physicians in the Netherlands who are specialized in amputation are aware of the possibility of an osseointegration prosthesis (OIP) and refer patients to our center for further assessment. More and more patients are becoming informed through social media and are asking their general practitioner for a referral to our clinic. In addition, a small group of people are referred by their surgeon. In recent years, an increasing number of patients are also sent to our center from abroad, sometimes spurred by our international website and scientific publications [https://www.radboudumc.nl/Zorg/Behandelingen/Pages/Osseointegration.aspx]. Patients additionally contact our clinic immediately after amputation to inquire about their eligibility for OIP treatment and to skip rehabilitation with a socket prosthesis. So far, our policy is to complete rehabilitation with a socket prosthesis and to only consider OIP after socket-related problems have been demonstrated [7].

**Fig. 1:**
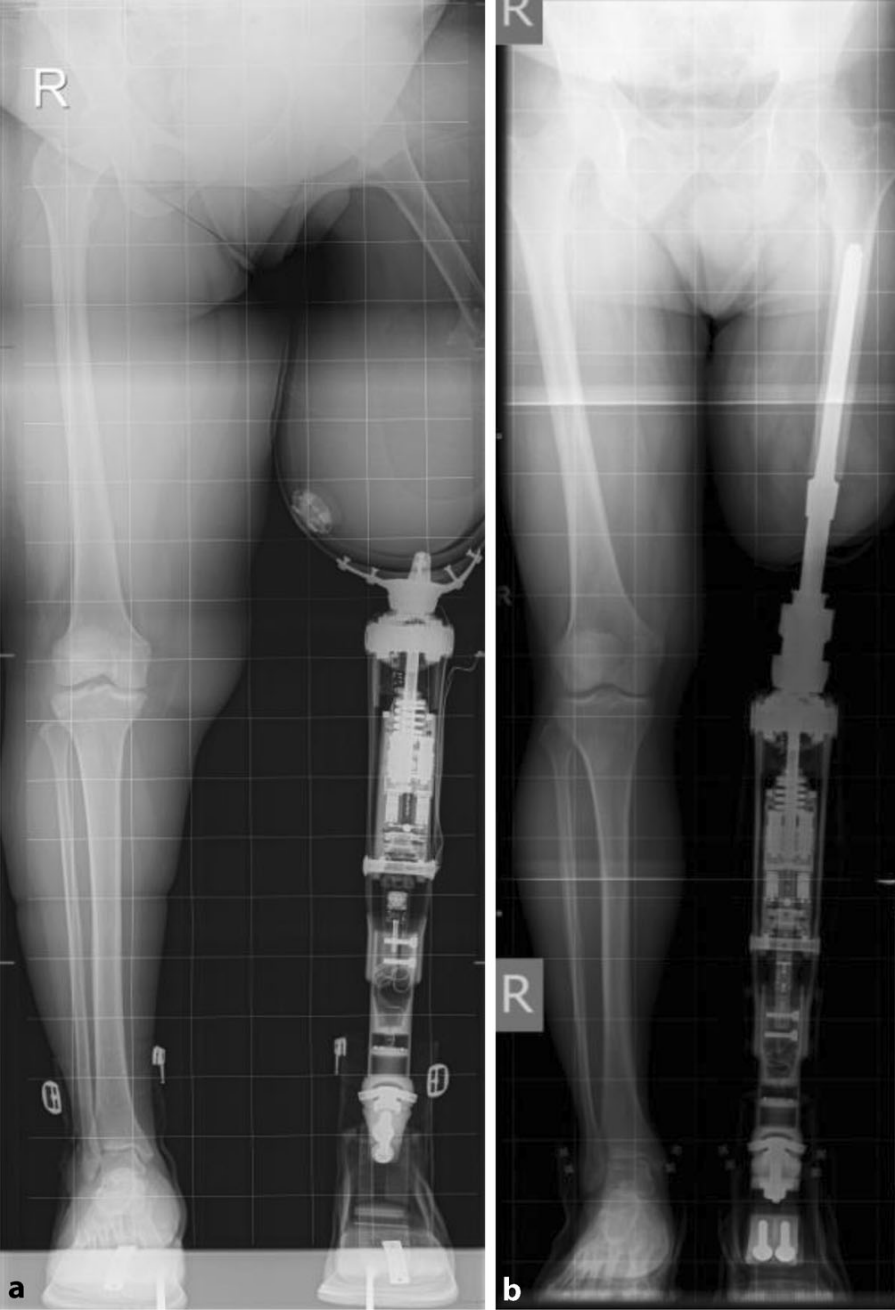
**a** Upright position with socket prosthesis and **b** with osseointegration prosthesis

Patients who are referred for OIP treatment are invited by our case manager to multidisciplinary group clinics. Prior to their visit, patient are asked to complete the Questionnaire for Persons with a Transfemoral Amputation (Q-TFA) [8]. The outpatient consultation that occurs once a month starts with standard radiographs collected in two directions, including a calibrated total view of both lower extremities, preferably in the upright position (Fig. 1a, b). In patients with transtibial amputation, a CT scan is performed to allow the design of patient-specific implants. Subsequently, patients and their companions are introduced to two OIP experts who were formerly treated at our center. These experts inform new patients about their experiences with the OIP treatment and what the new patients can expect regarding living with an OIP. Following the agenda of that day, a plenary presentation is performed for the entire group of OIP candidates by the surgeon and rehabilitation physician, in which all the details of the OIP treatment are discussed interactively. Finally, individual consultations with the OIP treatment team are performed, including a complete assessment of medical history, a physical examination, the Q‑TFA, and radiography to reach a consensus about the indication for OIP treatment based on shared decision making. The OIP treatment team includes an orthopedic surgeon, a rehabilitation physician, a physiotherapist, and an orthopedic technician. When a patient’s medical history reveals a psychiatric history, a clinical psychologist is also consulted to assess the patient prior to inclusion.

In 2009, OIP treatment was still associated with a risk of osteitis or osteomyelitis, so we only included patients with a lower limb amputation caused by trauma or a tumor in OIP treatment because we assumed that the patients in this category would have a relatively low risk of infection. Evaluation of the first 84 patients with an OIP in this low-risk category confirmed that the risk of osteitis or osteomyelitis was low [1]. In 2014, we decided to also include patients with lower limb amputation due to peripheral vascular disease and/or diabetes to qualify for an OIP. Future evaluation will show whether OIP treatment is safe in these patients with a higher average age, comorbidity and an increased risk of infections. The health benefits of an OIP in this category of patients are probably high because it is known that enhanced mobility is associated with higher life expectancy [5].

## Osseointegration systems

There are currently two registered OIP systems: the OPRA system (Integrum®) [4] and the Endo-Exo/ILP system (Eska/OrthoDynamics/Permedica) [6]. The OPRA system is derived from dental implants and consists of a titanium intramedullary implant (fixture) with a length of 80 mm that is connected to a transcutaneous abutment. The cylindrical fixture is inserted into the marrow cavity of the femur as a screw and needs 6 months for proper osseointegration [9]. The Endo-Exo/ILP system consists of a 140 to 180 mm-long, slightly curved chromium–cobalt–molybdenum or titanium stem with a macroporous or roughly coated surface that is hammered to be press fit in the medullary cavity and that allows loading within 6–8 weeks [3]. Full weight bearing is already possible after 8–12 weeks with the Endo-exo/ILP system compared with the OPRA system, which allows for full weight bearing after 6–12 months [13]. Because of the shorter osseointegration/rehabilitation period, we chose the Endo-Exo/ILP system for procedures performed in the Netherlands. The surgical procedure includes two operations [2]: in the first operation, the intramedullary implant is inserted in combination with a stump revision, and 6–8 weeks later, the second operation is scheduled for insertion of the transcutaneous component (dual cone adapter).

## Implant design

**Fig. 2:**
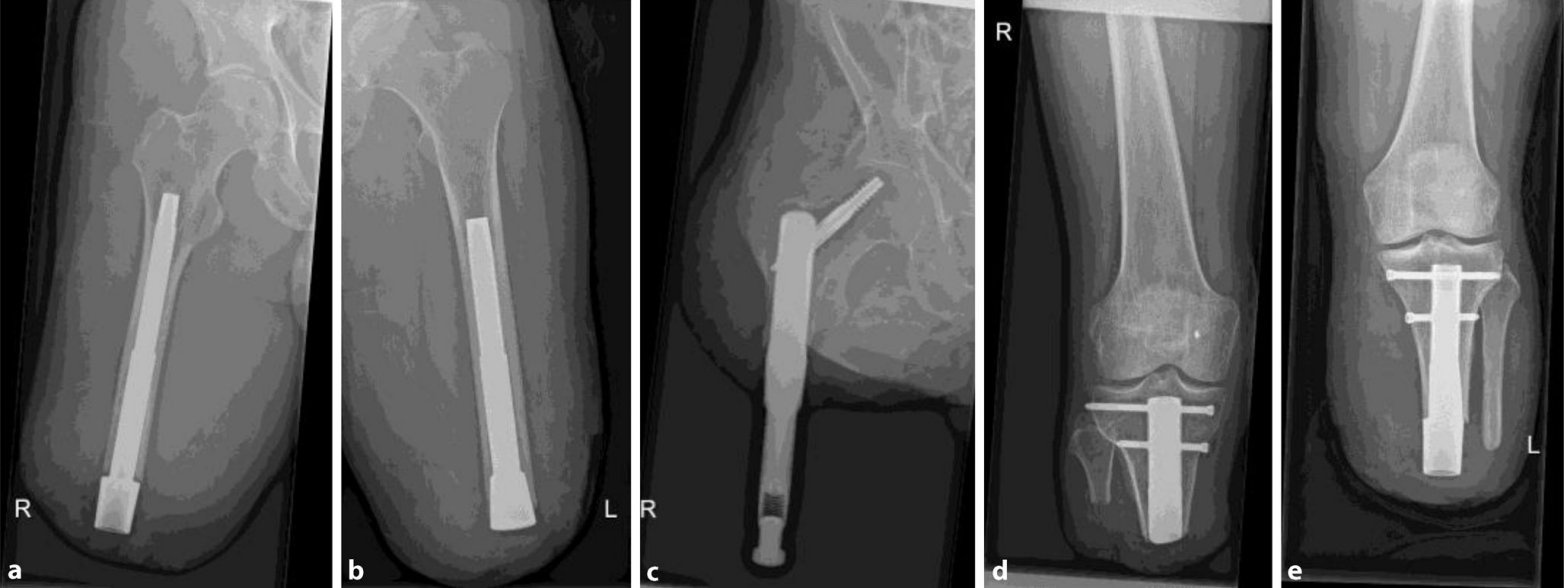
Examples of various implants available: **a** OPL type A with a distal niobium polished extramedullary head, **b** OPL type B with an intramedullary distal head, **c**, **d**, **e** custom-made implants with macroporous 3D mesh coating for accelerated osseointegration

The original chromium–cobalt–molybdenum cast stem in the Endo-exo/ILP system is covered with a macroporous coating of 1.5 mm thickness (tripods), reducing the core diameter of the femoral stem by 3 mm. After breakage of two Endo-Exo/ILP stems within 3 years after implantation, in 2015, we decided to proceed with a newly designed forged titanium implant system (OPL) manufactured with a thin plasma-sprayed coating and a standard length of 160 mm (Permedica, Sydney Australia). As for the chromium–cobalt–molybdenum stem, there is a distal niobium polished extramedullary head (OPL type A, Fig. 2a). For distal transfemoral amputations, there is also an intramedullary distal head (OPL type B, Fig. 2b). For patients with femur remnants of less than 160 mm (Fig. 2c) and for patients with transtibial amputation, we use custom-made implants with locking screws for primary stabilisation. These short implants are covered with a 0.5 mm macroporous 3D mesh coating for rapid osseointegration (Fig. 2d, e). Custom-made implants are designed in collaboration with engineers from the Radboud University Medical Centre 3D Laboratory (Nijmegen, The Netherlands) and physicians of the osseointegration team based on standard CT scans applied in a graphic design program. The design is then fabricated with a titanium 3D printer for further processing and coating.

## Preoperative planning

For patients after midshaft or distal transfemoral amputation, we analyze calibrated digital radiographs using orthopedic software to calculate the dimensions of the femoral implant (IMPAX Client AGFA, Rijswijk, The Netherlands). From standing full-leg-length radiographs, we determine whether the femoral remnant is to be shortened for implantation of the femoral stem. Based on the limb subject to the axis of rotation of the OIP, the OIP is brought flush with the axis of rotation of the knee on the side that was not amputated. The minimum construction height of the Endo-Exo/ILP system, including the transcutaneous dual cone adapter component and the external connector, is 160 mm. Calculated from the contralateral medial knee joint, knee or distal transfemoral amputations have to be shortened 160 mm at most.

## Surgical protocol

The OIP system is applied via two operations in the Netherlands. Both operations are carried out in accordance with the international standard orthopedic protocol used in hip and knee replacements, including perioperative intravenous antibiotic prophylaxis. The first operation includes an adjustment of the stump, with reduction of the soft tissue and possible shortening of the femur. Then, the medullary canal is opened and is reamed and rasped to the desired diameter in a retrograde manner. After selecting the correct implant size, the press-fit coated stem is inserted by gentle hammering. Primary stability is achieved by the slight curvature of the implant within the anatomical antecurvation of the femoral shaft in type A implants. Tibial implants and patients specific implants for short femoral remnants, primary stability is ensured by locking screws. Prior to the final insertion, the dorsal and ventral muscle groups of the thigh are connected to the distal femoral cortex with sutures to construct a myodesis. The future stoma site is prepared by removal of subcutaneous adipose tissue, and the skin is closed. All patients are dismissed on postoperative day three or four. In the period up to the second operation, no socket prosthesis can be used to optimize the wound healing. Mobilization must occur using crutches or a wheelchair. The artificial leg that was previously connected to the socket is collected by the orthopedic technician to mount the click safety connector for the osseointegration system (OTN Wijchen, The Netherlands). The second operation is planned for 6–8 weeks after the implantation of the intramedullary femoral stem and includes a small procedure that involves the creation of a stoma by cutting a circular (diameter of 20 mm) skin incision at the location of the head of the femoral stem. The transcutaneous component (dual cone) of the OIP system is placed in the head of the femoral stem and fixed with a locking screw.

**Fig. 3:**
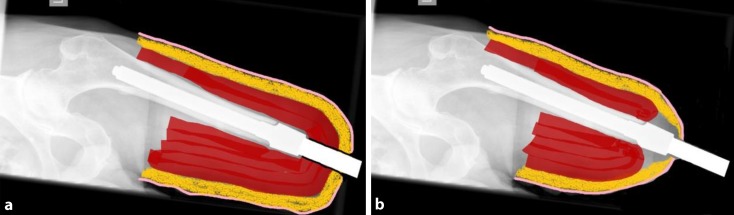
**a** Schematic representation of a ‘wet’ stoma, **b** schematic representation of a ‘dry’ stoma

Until 2012, we did not remove subcutaneous fat when preparing the stoma, and certain patients needed surgical refashioning of the stoma because it produced discharge due to mechanical friction between the soft tissue and the transcutaneous component (Fig. 3a). This discharge had to be collected with gauze, which was fixed with a silicone cap to prevent loss of moisture via contact with clothing (‘wet’ stoma). In an attempt to prevent these secondary refashioning effects and also to simplify stoma care, we adjusted our technique to include the construction of a myodesis at stage one as well as removal of subcutaneous fat to prepare the stoma (Fig. 3b). As a result, instead of the transcutaneous component of the system being introduced through the muscle and subcutaneous fat layers, only skin covers the intramedullary stem. This approach has resulted in a shorter range of the transcutaneous component in the soft tissue, resulting in less pain, less abscess formation, and lower production of discharge from the stoma (‘dry’ stoma). Future research should determine whether this surgical adjustment will have the desired effect.

Analogous to developments in dental implantology, there are also initiatives for femoral and tibial osseointegration systems to be implemented via one operation (single-stage method) [14, 15]. This approach, of course, has an important advantage in that there is only one surgery needed, instead of two, with a reduction of treatment time. In certain (tibia) cases, the soft tissue covering the stump is so thin that treatment in two stages is technically not possible. In those cases, the placement of the transcutaneous component, usually occurring in stage two, is performed in the first operation. The original idea for performing the OIP placement in two stages is to allow bony ingrowth of the femoral stem under sterile conditions. Adequate osseointegration of the implant prevents any infection from ascending from the stoma and entering the marrow cavity. In the case of single-stage procedures, the transcutaneous component of the OIP system is theoretically under nonsterile conditions during the osseointegration period, with a consequently possibly higher risk of osteomyelitis and septic loosening. To date, standard OIP treatment is carried out in two stages at our clinic. Further research will indicate whether single-stage OIP treatment is safe enough to offer as standard procedure.

## Rehabilitation protocol

The first session of rehabilitation following OIP treatment starts one week after the second operation. The rehabilitation is conducted by our multidisciplinary team twice a week with groups of 6–8 patients. The osseointegration rehabilitation team includes a rehabilitation physician, a physical therapist, an occupational therapist, and a prosthetic technician. Patients with a psychiatric history also receive cotherapy from a clinical psychologist, if needed. During the first session, the transcutaneous component of the OIP system is attached to the external prosthesis by means of a click safety adapter by the prosthetic technician, with adjustment and ultimate alignment at a later stage during rehabilitation. The physical therapist provides instructions for transitioning from partial weight bearing to walking with full loading, with symmetrical walking as the ultimate goal [11]. The occupational therapist provides instructions for daily stoma care. The rehabilitation physician supervises the entire rehabilitation and treats initial stoma complaints with prescriptions of painkillers or antibiotics, if needed. In cases of a tibial OIP, the duration of the total rehabilitation is approximately 4 weeks. In femoral cases, the rehabilitation is at least 4 weeks long. An interlude is initiated if muscle pain or limited muscle strength is an obstacle to further progression to full loading. In the interlude, patients walk with two crutches and cover walking distances at their own speed, depending on pain. The rehabilitation sessions are resumed when the patients are able to walk approximately 50–100 m with minimal support from the crutches. This part of the rehabilitation program focuses on walking longer distances without crutches and optimizing gait symmetry. For rehabilitation after OIP treatment, pain is typically in the distal stump, at the site of the myodesis. Only when the myodesis is very strong can the stump muscles optimally stabilize the femur, with gradual disappearance of the pain in the muscles and attachments. In most patients, it takes approximately one year after implantation to reach that desired level [16].

## Pre–post outcome measures

To evaluate functional outcomes in a systematic manner both preoperatively and at 6 months and 1, 2, 5, and 10 years after OIP placement, specific instruments are used to evaluate performance. The Q‑TFA is used to evaluate the prosthesis wearing time, mobility, and prosthesis-related quality of life [8]. Activities are assessed with the timed up and go test and the 6‑minute walk test. For all patients, the pre–post outcome data and complications are recorded in a certified web-based data management system (https://nl.castoredc.com) [12]. Standard conventional radiographs are used to assess bone remodeling at 1 and 2 years after OIP surgery.

## Revision of OIP system

The intramedullary component of the OIP system has to be replaced in cases of (a)septic loosening or implant breakage [1]. Similar to the replacement of a hip or knee prosthesis, it is technically possible to remove the intramedullary component of an OIP system and to replace it with a new implant. In such a revision, the distal head is sawed off the intramedullary component and subsequently removed with an oversized corer inevitably removing a small amount of cortical bone from the inner cortical wall. Research by our group has shown that periprosthetic cortical bone increases over the years, which facilitates surgical removal of the stem [10]. To allow the cortical wall to heal and to optimize osseointegration potential, we tend to wait 6 months to redo the stem implantation.

## Future developments

At this time, OIP treatment is applied to patients with problems that are related to the prosthesis socket [12]. Based on the favorable results with regard to security, mobility and quality of life, we expect an increasing number of patients eligible for OIP treatment to be treatable simultaneously with or directly after amputation, without socket adjustments. Moreover, patients with only slight socket-related complaints and patients who demand high-performance prosthesis will be eligible for OIP treatment. In addition, more patients with a lower limb amputation due to peripheral vascular disease and/or diabetes will be eligible for OIP treatment. The development of a standard tibial OIP system, rather than a labor- and time-intensive custom-made osseointegration system, will improve the quality of care and reduce healthcare costs. More and more people with transtibial amputation will qualify for an OIP in the future. Our osseointegration group is currently developing a standard tibial implant that meets the qualification for European Conformity (CE marking). In addition, the femoral OIP system will be further optimized so that the standard implant can also be used in patients with a shorter femur remnant.

## Practical conclusion

- Osseointegration prostheses (OIP) can be safely used in patients having problems related to the prosthesis socket.
- The associated risk for osteitis and osteomyelitis is low in patients with amputation due to trauma or tumor. The risk in other groups of patients (e.g., peripheral vascular disease, diabetes) is being evaluated.
- Standard length (140 or 160 mm) implants are available for transfemoral amputations; custom-made implants can be made for shorter remnants or for transtibial amputations.
- Total rehabilitation time approximately 4 weeks; interludes taken as needed due to muscle pain or inadequate muscle strength.
- Patients with OIP have improved mobility and quality of life.
